# Unveiling the Mysteries of Long COVID Syndrome: Exploring the Distinct Tissue and Organ Pathologies Linked to Prolonged COVID-19 Symptoms

**DOI:** 10.7759/cureus.44588

**Published:** 2023-09-02

**Authors:** FNU Sapna, FNU Deepa, FNU Sakshi, FNU Sonam, FNU Kiran, Raja Sandeep Perkash, Ahmed Bendari, Anish Kumar, Yusra Rizvi, FNU Suraksha, Giustino Varrassi

**Affiliations:** 1 Pathology and Laboratory Medicine, Albert Einstein College of Medicine, Bronx, USA; 2 Internal Medicine, Ghulam Muhammad Mahar Medical College, Sukkur, PAK; 3 Internal Medicine, Peoples University of Medical and Health Sciences for Women, Nawabshah, PAK; 4 Pathology and Laboratory Medicine, Dr. Ziauddin Hospital, Karachi, PAK; 5 Medicine, Mustafai Trust Central Hospital, Sukkur, PAK; 6 Pathology, University of Missouri School of Medicine, Columbia, USA; 7 Neurosurgery, Albert Einstein College of Medicine, Bronx, USA; 8 Pathology and Laboratory Medicine, Lenox Hill Hospital, New York, USA; 9 Internal Medicine, Dow University of Health Sciences, Karachi, PAK; 10 Internal Medicine, People University of Medical and Health Science for Women Nawabshah, Nawabshah, PAK; 11 Pain Medicine, Paolo Procacci Foundation, Rome, ITA

**Keywords:** investigating unique associations, long-term covid-19 symptoms, tissue and organ pathologies, pathological signatures, long covid syndrome

## Abstract

The ongoing battle against the coronavirus disease 2019 (COVID-19) pandemic has encountered a complex aspect with the emergence of long COVID syndrome. There has been a growing prevalence of COVID-19-affected individuals experiencing persistent and diverse symptoms that extend beyond the initial infection phase. The phenomenon known as long COVID syndrome raises significant questions about the underlying mechanisms driving these enduring symptoms. This comprehensive analysis explores the complex domain of long COVID syndrome with a view to shed light on the specific tissue and organ pathologies contributing to its intricate nature. This review aims to analyze the various clinical manifestations of this condition across different bodily systems and explore potential mechanisms such as viral persistence, immune dysregulation, autoimmunity, and molecular mimicry. The goal is to gain a better understanding of the intricate network of pathologies contributing to long COVID syndrome. Understanding these distinct pathological indicators provides valuable insights into comprehending the complexities of long COVID and presents opportunities for developing more accurate diagnostic and therapeutic strategies, thereby improving the quality of patient care by effectively addressing the ever-changing medical challenge in a more focused manner.

## Introduction and background

Our understanding of viral diseases and their impact has been challenged by the advent of the novel coronavirus disease (COVID-19), which is caused by the severe acute respiratory syndrome coronavirus 2 (SARS-CoV-2). In addition to the immediate difficulties brought on by the acute stage of the illness, a puzzling phenomenon known as "long COVID" has drawn the interest of researchers, clinicians, and public health specialists alike. Long COVID poses a challenging problem that calls for careful examination. It is characterized by a range of persistent symptoms that last far beyond the acute phase of infection. The goal of this narrative review is to thoroughly examine the complex picture of protracted COVID, highlighting its prevalence, variety of clinical presentations, and the urgent need to understand its underlying pathology.

A separate clinical entity known as long COVID, also known as "post-acute sequelae of SARS-CoV-2 infection" (PASC), has arisen, and its scope extends well beyond acute respiratory distress. Although COVID-19 was initially thought to be a respiratory condition, it has since been discovered to affect numerous organ systems and cause a variety of symptoms that are not just limited to the pulmonary area. While many people recover within a few weeks of the initial infection, a sizeable segment continues to experience ongoing, frequently incapacitating symptoms that affect their quality of life. There are several different manifestations of extended COVID symptoms. Deep exhaustion, cognitive issues commonly referred to as "brain fog," shortness of breath, joint and muscle discomfort, palpitations, and a slew of neurological symptoms like headaches and impaired sensory perception are among the most frequently reported ones [1]. The diversity and complexity of these symptoms make it difficult to categorize them, which emphasizes the importance of having a thorough grasp of the underlying disease mechanisms.

The problem of determining the prevalence of extended COVID is crucial because it affects resource allocation and healthcare planning. Due to a number of issues, such as different definitions of long COVID, inconsistent methodology, and differing follow-up times among research, it is challenging to obtain accurate estimates of the percentage of COVID-19 survivors who exhibit permanent symptoms. However, new research indicates that protracted COVID is widespread, with a considerable number of people still exhibiting symptoms even after moderate or silent initial infections [2]. For instance, Sudre et al.'s prospective investigation discovered that among people with confirmed COVID-19, almost 13.3% reported suffering symptoms that lasted longer than 28 days, and 4.5% reported symptoms that lasted longer than eight weeks [3]. Of note, 76% of those who recovered from COVID-19 suffered at least one lingering symptom after six months, according to Huang et al.'s findings in another study [4]. These findings highlight the necessity of identifying the underlying mechanisms of extended COVID in order to alleviate the suffering of those who are affected and to guide management and mitigation methods. Deeply troubling issues concerning the underlying mechanisms causing chronic COVID are raised by the symptoms' persistence long after the acute period of infection. This phenomenon is under close scientific investigation because its pathophysiology is still unknown.

Long COVID is a mystery that needs to be solved, and doing so has important clinical and public health ramifications. The most important priority in terms of managing and treating affected persons is identifying the pathophysiological underpinnings of extended COVID. Given the vast range of symptoms that COVID might present with, from respiratory to neurological, a thorough knowledge of the underlying mechanisms is essential for designing effective therapeutic strategies. For instance, immunomodulatory medications may present prospective options for symptom relief if immunological dysregulation is found to be a major contributor. Deciphering the complex web of protracted COVID may also help us throw light on the broader topic of post-viral disorders and immunological dysregulation. Long COVID, though a novel and distinct manifestation, has features in common with other post-viral syndromes, including post-acute sequelae of various viral infections and myalgic encephalomyelitis/chronic fatigue syndrome (ME/CFS). We may gain a deeper understanding of the mechanisms of protracted COVID and the persistence of symptoms in these diseases by conducting research into these mechanisms.

A thorough understanding of extended COVID could also have an impact on public health policies and plans. Effective COVID prevention and management, by lowering the number of people needing extended medical care and support, could reduce the burden on healthcare systems. Furthermore, public health messaging, treatment strategies, and immunization efforts should all benefit from having a good grasp of the risk factors associated with the growth of protracted COVID. The mystery of long COVID is a difficult problem that requires careful examination. With a focus on long COVID's prevalence, variety of clinical symptoms, and the pressing need to understand the underlying pathogenic mechanisms, this narrative review seeks to navigate the complex terrain of the disease. Our objective is to enhance awareness about this issue as researchers work to solve the complex riddle of extended COVID, ultimately resulting in better management, treatment, and prevention methods for those who are impacted globally.

## Review

Methodology

Literature Search and Selection 

A systematic literature search across several databases was performed in order to fully understand the distinct tissue and organ diseases connected to chronic COVID-19 symptoms. The main goal was to find pertinent studies that shed light on the complex mechanisms underlying persistent symptoms associated with long COVID and offered insights into the pathological basis of the disease. The methods used for this narrative review ensured that the pathological hallmarks connected to extended COVID syndrome were thoroughly and methodically investigated. This review seeks to provide a summarized overview of the many tissue and organ diseases that contribute to the ongoing symptoms experienced by people with extended COVID by utilizing stringent search methodologies and distinct inclusion criteria.

Inclusion and Exclusion Criteria

Studies covering the COVID-19 epidemic and its aftermath that were published between January 2020 and August 2023 were taken into consideration. Studies examining tissue and organ diseases linked to extended COVID that involved human participants were also included. To ensure a thorough synthesis of the available data, original research articles, case studies, clinical trials, and systematic reviews were taken into account. Studies that mainly examined acute COVID-19 infection and its immediate effects were excluded because the goal of this investigation was to look into the long-term manifestations of COVID-19. To ensure the relevance of the synthesized information, studies without a distinct focus on the pathological etiology of extended COVID were also excluded.

Sourcing From Databases and Search Terms Used

To find pertinent research on the pathological indicators of long COVID syndrome, a thorough search approach was developed. To make sure the entire body of the literature was covered, numerous databases were methodically searched. The search utilized the databases PubMed/MEDLINE, Embase, Scopus, Web of Science, and Google Scholar. The Medical Subject Headings (MeSH) terms were combined with pertinent keywords to create the search strategy. The keywords included terms and phrases such as "long COVID," "post-acute sequelae of SARS-CoV-2 infection," "pathological basis," "tissue pathology," "organ involvement," and associated terms. The search phrases were modified to fit each database's unique syntax and indexing rules. To bring focus to the search and guarantee accuracy, Boolean Operators (AND, OR) were used. To find the most pertinent and latest studies, the search syntax was modified to meet the needs of each database. A large selection of articles was found following the initial search. We eliminated duplicate articles and checked the relevance of the titles and abstracts of the rest of the articles. Then, based on the inclusion and exclusion criteria stated above, the full texts of any possibly pertinent articles were evaluated. When full texts were not available, attempts were made to obtain them through interlibrary loans or through getting in touch with the writers directly.

Data Extraction and Synthesis

To obtain the most relevant information from our investigations, data extraction was done methodically. Study design, sample size, participant demographics, techniques for evaluating tissues and organs, pathological findings, and significant conclusions on the distinctive pathological signs of extended COVID were all included in the retrieved data. The hierarchical organization of the retrieved data made it easier to synthesize and compare the results of various research studies.

Ethical Considerations 

Since this review only used publically available sources, ethical issues involving the use of human subjects or tissue were not relevant. The study complied with the standards and precepts of scholarly rigor and academic honesty.

Clinical manifestations of long COVID syndrome

The post-pandemic era has brought forth a complex medical phenomenon called "long COVID," which presents a multitude of clinical manifestations. As the COVID-19 pandemic progressed, it became increasingly clear that the pathophysiological effects of the SARS-CoV-2 virus extend beyond the initial phase of infection. Long COVID, also known as PASC, encompasses a diverse range of enduring symptoms that have the potential to impact various organ systems within the body. This article explores the comprehensive clinical manifestations of long COVID, providing insights into the prevalent symptoms, the diverse range of presentations, and the notable endurance of these symptoms.

Description of Frequently Experienced Long-Term COVID-19 Symptoms

The long COVID syndrome encompasses a diverse range of persistent symptoms that extend beyond the initial phase of the viral infection. It is important to recognize that long COVID symptoms can exhibit significant variation among individuals. However, certain recurring manifestations have emerged as characteristic features of this condition. Symptoms of fatigue and malaise have been reported. Profound fatigue is a highly prevalent and incapacitating symptom frequently reported by individuals who have experienced long COVID. Many individuals frequently report experiencing significant fatigue, even when engaging in minimal levels of physical or cognitive activity. The presence of fatigue may be accompanied by a widespread feeling of malaise and a decreased ability to participate in previously habitual activities [5]. Cognitive impairments, commonly known as "brain fog," are frequently reported by individuals with long COVID. This encompasses challenges related to concentration, memory retention, attention span, and multitasking abilities. The presence of cognitive impairments can have a substantial impact on an individual's daily functioning and overall quality of life [6].

Respiratory symptoms commonly observed in individuals with long COVID include shortness of breath, persistent cough, and chest discomfort. Even in individuals who did not exhibit severe respiratory symptoms during the acute phase, the persistent effect on lung function can contribute to continued respiratory distress [7]. Musculoskeletal pain refers to discomfort or pain that affects the muscles, bones, ligaments, tendons, or other structures. Long COVID survivors commonly experience joint pain, muscle aches, and general musculoskeletal discomfort. The pain is frequently characterized as migratory and has the potential to impact various regions of the body [8]. Cardiovascular symptoms such as palpitations, chest pain, and fluctuations in heart rate have been reported in individuals experiencing long COVID. Some individuals may experience a medical condition known as postural orthostatic tachycardia syndrome (POTS), which can result in feelings of dizziness upon assuming an upright position [9].

Long COVID patients may experience gastrointestinal disturbances such as nausea, diarrhea, and abdominal pain on occasion. The gastrointestinal symptoms may continue for an extended period of time even after the acute phase of infection is over [10]. They also experience neurological symptoms. Long COVID survivors have reported a range of neurological symptoms, such as headaches, dizziness, altered sensory perception, and neuropathic pain. The neurological manifestations observed in this study underscore the potential implications of the virus on the nervous system [11].

The Variability of Symptoms and Their Longevity

While there are certain common symptoms of long COVID that are observed among individuals, it is crucial to highlight the significant diversity in how these symptoms manifest. The diverse range of symptoms and their various combinations render long COVID a complex and formidable condition to both characterize and manage. An individual may present with a predominance of neurological symptoms, while another individual may primarily exhibit respiratory or musculoskeletal issues. In addition, the enduring presence of symptoms is a distinguishing feature of long COVID. While it is observed that certain individuals may undergo a gradual resolution of symptoms over a span of several months, some others continue to face persistent symptoms for an extended period, sometimes exceeding six months [12]. The extended duration of this condition presents a challenge to the conventional understanding of recuperation from viral infections, underscoring the necessity for a more sophisticated and all-encompassing approach to healthcare.

The mechanisms responsible for the continued presence of symptoms are currently the subject of extensive research. The persistence of symptoms of long COVID can be attributed to various factors, including immunological dysregulation, ongoing viral activity, autoimmunity, and the potential influence of psychological factors [13]. Understanding these mechanisms is crucial for the advancement of targeted interventions and therapies. The clinical manifestations associated with long COVID encompass a wide array of symptoms that affect various organ systems. The effects of this condition can have a significant and long-lasting impact, ranging from severe fatigue to neurological disturbances. The presence of diverse symptom manifestations highlights the intricate nature of long COVID, while the enduring nature of these symptoms poses a challenge to our comprehension of the recuperation journey. As researchers endeavor to unravel the complex pathophysiology associated with long COVID, there is an optimistic outlook that enhanced understanding will facilitate the development of more efficacious strategies for the management and support of individuals affected by this enigmatic syndrome.

Tissue and organ involvement in long COVID

The enduring presence of symptoms in individuals with long COVID, which is marked by a wide range of clinical manifestations, highlights the complex engagement of multiple organ systems. The objective of this comprehensive investigation is to analyze the pathophysiological foundation of long COVID, providing insights into the potential involvement of tissues and organs, as well as the underlying mechanisms that contribute to these enduring symptoms.

Respiratory System

The respiratory system is notably affected by long COVID, as individuals often continue to experience lingering respiratory symptoms even after the acute infection has resolved. The symptoms encompass persistent shortness of breath, chest discomfort, and cough [14]. There is a growing body of evidence indicating that the respiratory system serves as both a site of initial infection and a crucial location for ongoing immune responses. The presence of pulmonary complications in individuals with long COVID can be attributed to various factors, such as the persistence of the virus in lung tissue and dysregulation of the immune system. According to research findings, individuals with long COVID may present persistent lung abnormalities on imaging modalities like CT scans, even after the resolution of their initial symptoms [15]. This gives rise to concerns regarding the possibility of pulmonary damage and fibrosis following a viral infection. Additionally, it is important to note that the immune response triggered during the acute infection has the potential to result in a prolonged inflammatory condition, which may lead to tissue damage and potential restructuring. The presence of fibrotic changes may exacerbate respiratory symptoms and impair lung function.

Cardiovascular System

The cardiovascular system is known to be significantly affected by long COVID, leading to various complications that have a profound impact on the well-being of patients in the short term as well as their long-term cardiovascular health. Based on available evidence, the virus has the ability to directly infect cardiac tissue, resulting in myocarditis and other related cardiac complications [16]. The susceptibility of the heart to SARS-CoV-2 infection is attributed to the presence of angiotensin-converting enzyme 2 (ACE2) receptors, which act as portals for viral entry. The cardiovascular implications of long COVID extend beyond the initial acute infection, as individuals have reported experiencing symptoms such as palpitations, chest pain, and exercise intolerance [17]. The symptoms are believed to be caused by various mechanisms, including persistent inflammation, dysfunction of the microvascular system, and potential dysregulation of the autonomic nervous system. The cardiovascular manifestations have significant implications, highlighting the necessity for ongoing cardiac monitoring and interventions to reduce the risk of cardiovascular disease.

Neurological System

The neurological symptoms observed in individuals with long COVID are diverse and include headaches, dizziness, altered sensation, and cognitive disturbances commonly known as "brain fog" [18]. The susceptibility of the central nervous system to viral invasion, in addition to systemic immune responses, may potentially contribute to the occurrence of these neurological manifestations. Neuroinflammation, which is characterized by increased levels of cytokines and chemokines, could potentially have a significant impact on the continuation of neurological symptoms. Recent research has brought attention to potential neurological disorders linked to long COVID, including neurodegeneration and demyelination [19]. The aforementioned findings highlight the extensive influence of long COVID on neurological well-being and give rise to apprehensions regarding the potential enduring cognitive and neurological ramifications. Gaining a comprehensive understanding of the underlying mechanisms responsible for these neurological symptoms has the potential to offer valuable insights into developing therapeutic interventions aimed at reducing their impact.

Immune System Dysregulation

The immune response to SARS-CoV-2 infection has a dual nature, as it serves the purpose of eliminating the virus while also playing a role in the development of long COVID. The condition is characterized by immune dysregulation, wherein certain individuals may exhibit a prolonged and dysregulated immune response resulting in chronic inflammation [20]. Immune cells, such as monocytes, macrophages, and T cells, have been identified as potential contributors to inflammation and potential tissue damage in multiple organs. The investigation of the potential correlation between immune dysregulation and autoimmunity is a prominent focus in the field of long COVID research [21]. The immune response elicited against the virus may inadvertently result in the targeting of host tissues, thereby causing persistent inflammation and associated symptoms. It is imperative to comprehend the complex interaction between the immune response, pathologies specific to tissues, and potential autoimmune mechanisms in order to formulate precise therapeutic approaches.

Gastrointestinal and Renal Involvement

Individuals with long COVID have reported experiencing gastrointestinal and renal symptoms, such as nausea, diarrhea, and renal dysfunction [22]. Gastrointestinal symptoms may potentially emerge due to the virus's direct impact on gastrointestinal epithelial cells, while renal involvement could be attributed to systemic inflammation and vascular dysfunction. The specific underlying causes of gastrointestinal and renal symptoms in long COVID are not yet fully understood. However, it is clear that the virus's systemic effects can impact multiple organ systems. The necessity for a thorough evaluation and effective treatment is underscored by the potential enduring effects of gastrointestinal and renal complications. The intricate interplay of tissue and organ involvement in long COVID highlights the multifaceted nature of this condition. The respiratory, cardiovascular, neurological, immune, gastrointestinal, and renal systems are interconnected, with each system potentially playing a role in the ongoing symptoms experienced by affected individuals. It is crucial to have a thorough comprehension of the pathological mechanisms that underlie these symptoms in order to develop precise interventions, deliver the best possible patient care, and minimize the enduring effects of long COVID on patients' well-being.

Mechanisms underlying long COVID pathologies

The phenomenon of long COVID continues to present challenges, both in terms of the wide range of clinical symptoms observed and the complex mechanisms that contribute to its pathogenesis. The objective of this comprehensive investigation is to analyze the complex interaction between viral persistence, immune dysregulation, and autoimmunity in the context of long COVID. This study aims to provide insights into their potential roles and implications for treatment strategies.

Comparison of Viral Persistence and Immune Dysregulation

The central focus of the discussion regarding the pathogenesis of long COVID revolves around the intricate relationship between enduring viral components and the dysregulation of the immune system. Certain individuals with long COVID may continue to display detectable viral RNA even after the initial phase of infection, prompting inquiries into the potential influence of viral persistence on the persistence of symptoms [23]. Nevertheless, it is important to note that the mere existence of viral genetic material does not automatically indicate ongoing viral replication or an active infection. Alternatively, it is possible that the observed phenomenon may be attributed to the presence of residual viral fragments that elicit immune responses. However, it is increasingly acknowledged that immune dysregulation plays a crucial role in the pathophysiology of long COVID. Certain individuals demonstrate an atypical immune response characterized by prolonged inflammation, cytokine storm, and modified immune cell functions [24].

The dysregulation of the immune system can sustain tissue damage and play a role in the continuation of symptoms. The complex correlation between viral persistence and immune dysregulation in long COVID continues to be a topic of active research. There is a possibility that viral components remain present in certain reservoirs, eliciting immune responses that can have both beneficial and harmful effects. Viral elements have the potential to function as antigens, thereby triggering immune responses that unintentionally attack host tissues and contribute to the progression of pathology [25]. Maintaining a well-balanced immune response is crucial in effectively resolving infections while simultaneously preventing the occurrence of excessive inflammation and subsequent tissue damage. There is emerging evidence indicating that autoimmunity may contribute to the persistence of long COVID symptoms. Autoantibodies, which are antibodies that specifically target the tissues of the body, have been identified in certain individuals experiencing long-term symptoms of COVID-19. This finding suggests the possibility of immune responses directed against self-antigens. [26]. This presents the intriguing potential for molecular mimicry, wherein immune responses elicited against viral antigens exhibit cross-reactivity with host tissues as a result of structural resemblances.

Autoantibodies that specifically target various organs and tissues have been detected in individuals with long COVID, encompassing the respiratory system, cardiovascular system, and nervous system [27]. For example, the presence of autoantibodies that specifically target components of the vascular endothelium may potentially contribute to the development of cardiovascular complications. In a similar vein, it is plausible that the presence of autoantibodies specifically targeting neuronal tissues may be responsible for the neurological symptoms that are commonly observed in individuals experiencing long COVID. The ramifications of autoimmunity in the context of long COVID are extensive. Autoimmune responses have the potential to sustain inflammation, contribute to tissue damage, and worsen the persistence of symptoms. Gaining a comprehensive understanding of the scope of autoimmunity in long COVID, its underlying mechanisms, and its impact on various organ systems may prove instrumental in deciphering the intricate nature of this condition.

Considerations for Treatment Strategies

The acknowledgment of viral persistence, immune dysregulation, and autoimmunity as potential mechanisms in long COVID carries substantial implications for the advancement of treatment strategies. Strategically designed interventions that specifically target each of these mechanisms have the potential to be effective in mitigating symptoms and enhancing patient outcomes.

Antiviral strategies: In the event that viral persistence is found to have a substantial impact, it may be worthwhile to investigate antiviral therapies that specifically target viral reservoirs. Nevertheless, it is crucial to maintain a delicate equilibrium when implementing these interventions in order to prevent any potential aggravation of immune dysregulation.

Immunomodulation: Approaches that seek to modulate the immune response, such as the use of anti-inflammatory agents or immune checkpoint inhibitors, have the potential to restore immune homeostasis and alleviate chronic inflammation.

Autoimmune interventions: In cases where patients exhibit signs of autoimmunity, there is potential for symptom relief through the use of therapies that specifically target autoantibodies or regulate autoimmune responses. However, it is imperative that these interventions are customized to suit the specific autoantigens implicated.

Multidisciplinary care: In light of the extensive involvement of multiple organs in long COVID, it is imperative to adopt a multidisciplinary approach that incorporates specialists from various fields. Collaborative endeavors can effectively facilitate a thorough evaluation of patients' clinical presentation, thereby enabling the implementation of focused interventions.

The mechanisms that contribute to the pathologies of long COVID are intricate and multifaceted, characterized by a delicate interplay between viral persistence, immune dysregulation, and autoimmunity. Although viral elements may continue to exist in certain reservoirs, the persistence of symptoms and tissue damage is influenced by immune responses and the potential for autoimmunity. Comprehending the complex interconnections among these mechanisms is crucial for the development of efficacious treatment strategies aimed at alleviating the distress experienced by individuals with long COVID and enhancing their long-term prognosis.

Diagnostic challenges and biomarkers

The diagnosis of long COVID syndrome poses distinct challenges due to its varied clinical presentations and the lack of a universally accepted definition. The purpose of this extensive investigation is to shed light on the complex diagnostic landscape of long COVID and examine potential biomarkers that may assist in identifying the underlying pathologies associated with this mysterious condition.

Challenges in the Diagnosis of Long COVID Syndrome

The condition known as Long COVID presents a range of enduring symptoms that frequently overlap, thereby presenting notable difficulties in the diagnostic process. In contrast to the diagnosable nature of acute COVID-19 through viral testing, the absence of a universally accepted standard for identifying long COVID presents challenges in its diagnosis. There are several factors that contribute to these diagnostic challenges. Establishing a consistent diagnostic criterion for long COVID patients is challenging due to the wide range of symptoms they experience. The symptoms encompass a wide range of manifestations, including fatigue, cognitive impairments, as well as respiratory and cardiovascular issues [28]. Several symptoms commonly associated with long COVID, including fatigue and cognitive impairments, exhibit similarities with symptoms observed in other medical conditions, such as ME/CFS and fibromyalgia. The occurrence of this overlap has the potential to result in misdiagnosis or delayed diagnosis [29].

In contrast to acute COVID-19, which can be diagnosed through viral tests, long COVID does not possess objective diagnostic markers. Imaging and laboratory tests frequently do not yield conclusive evidence of the condition, resulting in a state of uncertainty. The determination of the duration of symptoms necessary to categorize an individual as experiencing long COVID is a topic of ongoing discussion and deliberation. The absence of consensus poses challenges to the implementation of standardized diagnostic approaches. In light of the diagnostic difficulties, researchers are currently engaged in the investigation of potential biomarkers that may offer valuable information regarding the underlying pathologies associated with long COVID. Biomarkers are quantifiable indicators, such as proteins or genetic markers, that have the ability to indicate the existence or advancement of a disease. The identification of dependable biomarkers has the potential to enhance diagnostic precision, inform treatment approaches, and foster a more comprehensive comprehension of the condition.

In the context of long COVID, inflammatory biomarkers such as C-reactive protein (CRP), interleukin-6 (IL-6), and tumor necrosis factor-alpha (TNF-α) have been investigated due to their association with immune dysregulation [30]. Increased levels of these markers may indicate persistent inflammation and could potentially assist in the diagnosis and monitoring of the condition. The identification of autoimmunity as a potential mechanism in long COVID has prompted research into the use of autoantibodies as diagnostic indicators. The identification of distinct autoantibodies linked to various organ systems can offer valuable insights into the specific tissues and organs that are affected [31].

Metabolomics and lipidomics encompass the thorough examination of metabolites and lipids within biological specimens. These methodologies have demonstrated modified metabolic profiles in individuals with long COVID, presenting potential biomarkers that may indicate underlying pathologies [32]. In individuals experiencing neurological symptoms, the utilization of neuroimaging biomarkers, such as structural and functional brain changes, may provide valuable insights into the neurological consequences of long COVID [33]. These biomarkers have the potential to distinguish neurological changes associated with long COVID from other medical conditions.

Cardiac Biomarkers: Patients experiencing prolonged symptoms of COVID-19 with cardiovascular manifestations may present with atypical levels of cardiac biomarkers, including troponin and brain natriuretic peptide (BNP). These biomarkers serve as indicators of potential myocardial damage or dysfunction [34].

The Future Outlook

The ongoing pursuit of precise biomarkers and diagnostic criteria for long COVID is motivated by the urgency to deliver suitable care for individuals impacted by this condition. The identification of dependable biomarkers has the potential to not only facilitate the diagnosis of long COVID but also contribute to treatment decision-making and enhance our understanding of the underlying mechanisms involved in this condition. Effective collaboration among clinicians, researchers, and healthcare institutions is imperative in addressing the diagnostic complexities associated with long COVID. As our comprehension of the condition progresses, it is anticipated that a comprehensive diagnostic approach encompassing clinical, laboratory, and imaging data will be imperative to ensure precise and prompt identification of individuals with long COVID. The process of diagnosing long COVID presents a significant challenge due to its wide range of symptoms, potential overlap with other medical conditions, and the absence of clear diagnostic criteria. The lack of objective markers exacerbates these challenges.

Nevertheless, the exploration of biomarkers shows potential in elucidating the fundamental pathologies associated with long COVID. Inflammatory markers, autoantibodies, metabolomics, neuroimaging, and cardiac biomarkers are among the potential indicators that may offer valuable insights into the underlying mechanisms of the condition. As scientific advancements continue, biomarkers have the potential to become invaluable instruments in the diagnosis, treatment, and comprehension of long COVID, ultimately enhancing the quality of life for individuals impacted by this intricate syndrome.

Therapeutic implications

As our understanding of long COVID deepens, the medical community faces a considerable challenge in effectively addressing the wide range of persistent symptoms associated with this condition. This comprehensive investigation examines the current treatment strategies for individuals with long COVID and emphasizes the significance of customizing interventions according to the underlying pathological mechanisms that contribute to this perplexing condition.

Evaluation of Current Treatment Approaches

The lack of a universally applicable treatment approach for long COVID reflects the complex nature of this condition. Although a definitive cure has not yet been discovered, various therapeutic approaches are currently being investigated with the aim of alleviating symptoms, enhancing the quality of life, and improving the overall well-being of patients.

Management of symptoms: A significant number of individuals with long COVID commonly encounter symptoms such as fatigue, cognitive impairments, pain, and breathlessness. The management of symptoms is a fundamental aspect of treatment, which includes the implementation of pain management techniques, cognitive rehabilitation, and the utilization of pacing strategies to effectively conserve energy [35].

Physiotherapy and rehabilitation services: Physical and occupational therapy are essential components in the management of musculoskeletal and functional impairments commonly associated with long COVID. Therapists customize exercise programs to systematically enhance endurance and muscular power [36].

Psychological support: The psychological impact of long COVID, which encompasses symptoms such as anxiety and depression, is substantial. Mental health interventions, such as cognitive behavioral therapy and mindfulness, have been found to offer effective coping strategies and emotional support [37].

Cardiovascular interventions: In the case of patients experiencing cardiovascular symptoms, it has been observed that specific interventions such as the administration of beta-blockers and the implementation of exercise programs have the potential to effectively address heart rate fluctuations and enhance cardiovascular fitness [38]. Pulmonary rehabilitation is a comprehensive program designed to improve the overall well-being and functional capacity of individuals with chronic respiratory conditions. Individuals suffering from long COVID and experiencing persistent respiratory symptoms may potentially derive significant advantages from participating in pulmonary rehabilitation programs. These programs are designed to enhance lung function, strengthen respiratory muscles, and improve overall physical endurance [38]. Immunomodulatory therapies may be considered in situations where there is notable immune dysregulation. These therapies, such as corticosteroids or biologic agents, aim to mitigate the inflammatory response [39].

Tailoring Treatments According to Underlying Mechanisms

As our comprehension of the underlying pathological mechanisms of long COVID continues to advance, the possibility of developing customized treatment strategies becomes apparent. Recognizing the wide range of mechanisms, symptoms, and organ involvement can inform the formulation of interventions that target specific facets of the condition.

Viral persistence and the potential use of antiviral therapies: In cases where individuals exhibit indications of persistent viral elements, it may be beneficial to consider the utilization of antiviral agents. These agents can be employed to specifically target reservoirs and restrict viral replication [40]. However, it is important to take into account the potential impact on immune responses and the intricate equilibrium between viral clearance and immune dysregulation.

The modulation of the immune system: The targeting of immune dysregulation may encompass various strategies aimed at modulating immune responses, restoring immune homeostasis, and mitigating chronic inflammation [38,39]. This may involve the utilization of immune checkpoint inhibitors or immunosuppressive agents in specific instances.

Autoimmunity and the potential of immunotherapies: Individuals exhibiting the presence of autoantibodies and clear indications of autoimmunity may potentially derive advantages from immunotherapies that specifically target autoantigens or modulate autoimmune responses [37]. This approach seeks to mitigate the tissue damage caused by autoantibodies.

Neurological and cognitive interventions: In patients experiencing neurological symptoms and cognitive impairments, it is possible to customize neurorehabilitation and cognitive therapies to target specific deficits [40]. These interventions have the potential to enhance cognitive function and contribute to an overall improvement in quality of life.

The customization of cardiovascular interventions to meet the specific needs of each patient, in addition to providing cardiopulmonary rehabilitation, has the potential to enhance heart function, increase exercise tolerance, and alleviate symptoms [41]. Effectively navigating the therapeutic landscape of long COVID necessitates adopting a comprehensive approach that recognizes the varied symptomatology, underlying mechanisms, and unique experiences of individual patients. Although a definitive cure has not yet been discovered, current treatment approaches provide opportunities for effectively managing symptoms and enhancing the overall well-being of patients. The advancement of long COVID therapeutics hinges on the personalized customization of interventions, taking into account the specific underlying pathological mechanisms. Clinicians can strive to develop targeted treatments that address the unique needs of each patient by acknowledging the intricate relationship between viral persistence, immune dysregulation, autoimmunity, and organ-specific pathologies. The collaboration of clinicians, researchers, and patients is crucial in developing a strategic plan for effective management and enhanced outcomes for individuals affected by this complex and mysterious syndrome.

Future directions and research gaps

As the scientific community endeavors to comprehend the intricacies of long COVID, the trajectory ahead is marked by a multitude of research pathways and collaborative efforts that hold the potential to enhance our comprehension of this enigmatic syndrome. This comprehensive study examines the future directions for long COVID research, highlighting key areas that require further investigation and emphasizing the importance of interdisciplinary collaboration in unraveling its complexities.

Identification of Areas Requiring Further Investigation

The complex characteristics of long COVID underscore the need for continuous research aimed at addressing various crucial inquiries and knowledge gaps:

Pathological mechanisms: Despite significant advancements, the exact pathological mechanisms underlying the diverse symptoms of long COVID remain elusive. Additional research is necessary to elucidate the complex relationship between viral persistence, immune dysregulation, autoimmunity, and pathologies specific to certain tissues [41]. The identification of dependable biomarkers for the diagnosis and monitoring of long COVID is of utmost importance. The objective of the research should be to authenticate established biomarkers and identify new indicators that demonstrate correlation with specific pathological mechanisms [42].

Treatment strategies: The development of effective treatment approaches that are customized to individual patients necessitates a comprehensive understanding of the underlying mechanisms involved. Conducting research on the effectiveness of antiviral agents, immunomodulatory therapies, and interventions aimed at addressing autoimmunity has the potential to provide valuable insights [43].

Long-term implications: The long-term effects of long COVID on patients' health are currently unclear. The research should prioritize the investigation of chronic organ damage, cognitive impairments, and overall quality of life beyond the acute phase [42].

Pediatric long COVID: The prevalence and impact of long COVID in children are subjects that warrant particular attention. Exploring the distinctive characteristics of long COVID in pediatric populations has the potential to provide valuable insights for the development of customized interventions and support measures [44]. The correlation between long COVID and vaccine responses necessitates further investigation. It is of utmost importance to conduct an investigation into the potential of vaccination to alleviate long COVID symptoms and its potential effects on underlying mechanisms [45].

The Significance of Interdisciplinary Research Collaboration

The complex nature of Long COVID highlights the importance of interdisciplinary collaboration in research. Collaborative endeavors among researchers, clinicians, epidemiologists, immunologists, neuroscientists, and other experts have the potential to bridge existing knowledge gaps and facilitate the development of a comprehensive understanding of the syndrome.

Holistic perspectives: Interdisciplinary collaboration facilitates a multifaceted approach to long COVID, thereby enhancing the scope and depth of insights. Insights from various disciplines can provide valuable understanding regarding the interrelatedness of symptoms and mechanisms. The research on long COVID encompasses a wide range of data types, such as clinical, molecular, and imaging data. Interdisciplinary collaboration plays a crucial role in facilitating the integration of various data sources, thereby enabling a more comprehensive analysis and interpretation of findings.

Targeted interventions: The collaboration between clinicians and researchers facilitates the development of treatment strategies that are informed by a comprehensive understanding of the underlying mechanisms. Tailoring interventions to individual patients can be significantly enhanced by incorporating insights from diverse disciplines. The process of translating research findings into clinical practice necessitates the collaboration and expertise of both researchers and clinicians. Collaborative endeavors play a crucial role in ensuring that research findings are relevant, feasible, and advantageous for the provision of patient care. The study design is characterized by its robustness. In order to effectively address the intricate nature of long COVID, it is imperative to incorporate insights from various disciplines during the design of comprehensive studies. The utilization of collaborative research design has the potential to enhance the validity of studies, mitigate biases, and improve the reliability of findings. The pursuit of understanding long COVID and creating effective interventions is a multifaceted undertaking that necessitates a collaborative and interdisciplinary approach. The identification of research gaps and the pursuit of future directions are crucial in addressing the uncertainties surrounding the underlying mechanisms, diagnostic criteria, treatment strategies, and long-term outcomes of long COVID. As professionals from various research and clinical disciplines converge, interdisciplinary collaboration plays a crucial role in navigating the unexplored realms of long COVID. This collaborative approach not only enhances patient care but also deepens our comprehension and sheds light on the intricate nature of this syndrome.

## Conclusions

This narrative review has shed light on key insights that contribute to a better understanding of the complex landscape of long COVID. These findings have significant implications for enhancing patient care. The review provides a comprehensive examination of the phenomenon known as long COVID, including its prevalence and the urgent necessity to understand its underlying pathological characteristics. The phenomenon known as Long COVID has emerged as a multifaceted syndrome characterized by a wide range of persistent symptoms that extend beyond the initial acute phase of infection. This narrative review explored the variability and enduring nature of these symptoms, emphasizing the difficulties they present in terms of diagnosis and treatment. By conducting a thorough examination of the respiratory, cardiovascular, neurological, immune, gastrointestinal, and renal systems, the review has successfully identified the various organ-specific pathologies that collectively contribute to the overall clinical presentation.

This review highlights the significant relationship between viral persistence, immune dysregulation, and potential autoimmunity as key factors contributing to the development of long COVID pathologies. The complex interaction between various factors not only extends the duration of symptoms but also initiates persistent inflammation, tissue harm, and potential complications mediated by autoantibodies. Based on these findings, the review highlights the utmost significance of accurately identifying dependable biomarkers and customizing treatment strategies to suit individual patients. Potential approaches to address the diverse symptoms and mechanisms of long COVID include symptomatic management, rehabilitation, immunomodulation, and organ-specific interventions. Moreover, the review highlights the importance of deciphering pathological signatures in order to advance patient care. By gaining a comprehensive understanding of the fundamental mechanisms, healthcare professionals are able to provide specific interventions that effectively alleviate symptoms, enhance the overall quality of life, and optimize long-term outcomes. We believe this narrative review offers a well-defined roadmap for the medical community. This roadmap aims to address the intricate nature of long COVID and provide a holistic approach to care for individuals affected by this complex syndrome.
